# Enhanced autopsy triage (EA-Triage) in drug-related deaths: integrating quick toxicological analysis and postmortem computed tomography

**DOI:** 10.1007/s12024-024-00819-2

**Published:** 2024-04-29

**Authors:** Lea Wold Kisbye, Annika Rickert, Jørgen Bo Hasselstrøm, Charlotte Uggerhøj Andersen, Henriette Askjær Lund, Marianne Cathrine Rohde, Lene Warner Thorup Boel

**Affiliations:** Department of Forensic Medicine, Aarhus University, Palle Juul- Jensens Boulevard 99, Aarhus N, 8200 Denmark

**Keywords:** Autopsy triage, Quick toxicological analysis, Postmortem computed tomography, Forensic toxicology, Cause of death, Forensic pathology

## Abstract

The objective was to assess the diagnostic accuracy of an enhanced autopsy triage (EA-Triage) setup consisting of postmortem computed tomography (PMCT), simulated quick toxicological analysis (sQTA), external examination, and case information in determining cause of death (COD) in persons with past or current use of illegal drugs (drug-related deaths). Information on drug-related deaths selected for medico-legal autopsy in 2020–2021 at the Department of Forensic Medicine, Aarhus University, Denmark, was analyzed retrospectively. The included cases underwent conventional autopsy, PMCT, and systematic toxicological analysis. A board-certified forensic pathologist, who was blinded to the internal examination and COD from the medico-legal autopsy, determined COD based on the EA-Triage setup. 154 cases with a median age of 40.6 years (range 17–70 years, 82% males) were included. The COD determined by medico-legal autopsy and that determined by EA-Triage matched in 113 cases (73%), including those with an unknown COD. EA-Triage and medico-legal autopsy determined unknown COD in 45 (29%) and 5 cases (3%), respectively. Excluding cases with an unknown COD, EA-Triage predicted COD in 109 cases (71%); of those, 72 (66%) had no unexplained case circumstances or suspicion of a criminal act. In these 72 cases, the CODs determined by EA-Triage and medico-legal autopsy matched in 71 cases (99%), and the sensitivity and specificity for detecting lethal intoxication were 100% and 90%, respectively. EA-Triage showed strong diagnostic accuracy for determining COD in drug-related deaths. This method may be suitable for enhancing preautopsy triage and guiding police investigations at an early stage.

**Supplementary Information:**

The online version contains supplementary material available at 10.1007/s12024-024-00819-2.

## Introduction

With the advancement and integration of diagnostic techniques in the field of forensics, the use of screening tools for preautopsy investigations or autopsy triage has been proposed [1, 2]. Minimally invasive screening methods, such as postmortem imaging and imaging-guided biopsy, have been explored, but toxicological analysis of postmortem blood and urine samples remains underexplored as a screening method. There is growing interest in using imaging methods to determine the cause of death (COD) prior to autopsy, which has led to discussions on the added value of subsequent autopsies [3, 4]. Extensive research on postmortem computed tomography (PMCT) as a screening tool has demonstrated promising diagnostic accuracy for COD in various subgroups of traumatic and nontraumatic deaths [5, 6]. PMCT was superior to autopsy in detecting skeletal injuries and in situ air, although agreement on COD determination in current research is variable [7, 8].

Deaths among individuals with a history of or current illegal narcotic drug use (drug-related deaths) constitute a large proportion of Danish forensic cases and are prone to poisoning. They are routinely autopsied due to the current Danish legislation requiring medico-legal autopsy for deaths related to illegal drug use [9]. Notably, the value of incorporating toxicological screening into preautopsy forensic investigations has not been extensively studied. Previous experience in the USA indicates that in cases of drug fatalities, autopsy or internal examination does not contribute to determination of a COD [10]. Instead, COD is often determined based on case information or toxicological analysis. Recent research suggests that combining PMCT with toxicological screening could improve the agreement of COD determination [11, 12]. However, conducting a full systematic toxicological analysis is time-consuming and currently unsuitable as a screening tool.

In Denmark, case selection for medico-legal autopsy occurs during a medico-legal inquest commonly conducted by a police officer with advice from a medical doctor affiliated with the Danish Patient Safety Authority [13]. The medico-legal inquest is mandated in cases of death related to criminal offense, suicide, accident, unwitnessed death, sudden unexpected death, occupational disease, malpractice, and death in police custody or when none of the above can be excluded with certainty. Based on information gathered from police reports, medical health information, and external examinations, the police officer decides whether a medico-legal autopsy is needed. Integrating diagnostic tools into this screening process has the potential to enhance the accuracy of preautopsy case selection. Advanced imaging techniques such as PMCT, combined with day-to-day toxicological analysis, could offer a fast and more comprehensive approach to autopsy triage [12]. PMCT is a noninvasive imaging technique that provides detailed visualizations of internal structures, while the development of quick toxicological analysis (QTA) for postmortem blood could aid in the identification of common drugs within 24–48 h of the investigation.

Therefore, this study aimed to explore enhanced autopsy triage (EA-Triage) by combining the capabilities of PMCT and simulated quick toxicological analysis (sQTA) with case information and external examination. The objective of this study was to assess the collective ability of these methods to accurately determine COD in a selected subgroup of drug-related fatalities. We hypothesize that the COD determined by EA-Triage can be comparable to that obtained from conventional autopsy.

## Materials and methods

### Study population

This study retrospectively analyzed information on drug-related deaths selected for medico-legal autopsies from 2020 to 2021 at the Department of Forensic Medicine, Aarhus University (AU), Denmark. All medico-legal autopsies were initiated through medico-legal inquests performed by four police districts in northwestern Denmark, covering 2.3 million inhabitants [14]. The study population of drug-related deaths was identified retrospectively at the Department of Forensic Medicine, AU, according to an in-house coding system based on the information gathered from the medico-legal autopsy report. The inclusion criteria were individuals with a history of current or past use of illegal drugs, as well as findings of illegal drugs either at the crime scene or through systematic toxicological analysis (STA). Persons who used cannabis alone were also included. Cases were selected regardless of the manner of death, but homicide cases were excluded. Medico-legal autopsies without PMCT or STA results or insufficient femoral blood (FB) for STA due to decomposition were also excluded.

### Medico-legal autopsy

All medico-legal autopsies were performed according to national guidelines by two doctors from the Department of Forensic Medicine, AU, one of which was a chief forensic pathologist. The department is certified according to international standards (ISO 17,020 for forensic pathology and ISO 17,025 for forensic chemistry). According to Danish legislation, medico-legal autopsies must be performed when the manner and/or COD are uncertain, when the death can be related to any criminal offense or suspicion of a criminal offense can be raised, or in suspected drug-related deaths [9, 13]. The medico-legal autopsy included a concise overview of the case information, findings from PMCT, thorough evaluations of both external and internal examinations, and ancillary tests, if requested (histology, STA, biochemistry, and microbiology). Finally, the forensic pathologist determined the manner of death and COD from the World Health Organization’s (WHO) International Classification of Diseases, Tenth Revision (ICD-10) according to national statistics guidelines [15, 16].

PMCT was performed prior to autopsy using a Canon Aquilion Prime SP 160 slice CT scanner (Canon Medical Systems, USA) using automatic exposure control (AEC) at 120 kV with collimation set to 0.5 × 80 mm. A full-body CT scan without contrast agent was performed with the deceased still in a body bag and without removing clothing. Afterward, one of two board-certified forensic pathologists trained in PMCT or a forensic radiographer evaluated the PMCT images. An experienced radiologist assigned to the department was available for consultation in all cases. PMCT reporting was performed according to a standardized template with detailed review of all organs, as well as an assessment of both skeletal and soft tissues (Online Resource 1).

STA consisted of an initial qualitative screening of postmortem femoral blood (FB), which was analyzed via ultra-performance liquid chromatography with high-resolution time-of-flight mass spectrometry (UPLC-QTOF) for more than 900 drugs and metabolites [17]. Positive screening results were verified and quantified by ultra-performance liquid chromatography with tandem mass spectrometry (LC‒MS/MS) [18, 19]. Ethanol and carbon monoxide were measured separately by headspace gas chromatography coupled to either a flame ionization detector or a mass spectrometer, respectively. A final assessment of the contributions of the analytical findings to the COD was conducted by consensus between two forensic chemists/clinical pharmacologists. This assessment was incorporated into the final autopsy report.

### Enhanced autopsy triage (EA-Triage)

To simulate an EA-Triage setup, one of two board-certified forensic pathologists was presented with a case summary, medical history, resuscitative treatment, external examination, the standardized template from the PMCT and sQTA results. The pathologists were blinded to the internal examination, the manner of death and the COD determined by the medico-legal autopsy.

Eighty-eight drugs and metabolites, chosen based on frequency of use in the population and findings related to drug-related deaths, plus ethanol and carbon monoxide were selected for inclusion in a quantitative manner in the sQTA. The sQTA was constructed for use in this study based on historical results from the toxicological laboratory. The sQTA included a toxicological evaluation of ethanol, carbon monoxide and the 88 chosen drugs and metabolites (Online Resource 2) from the STA. The remaining compounds from the more than 900 compounds detected by the current screening procedure were included in the sQTA in a qualitative manner, i.e., as detected or not detected. The development of a method encompassing these 88 drugs and metabolites, ethanol, and carbon monoxide that could present results of both quantitative and qualitative value within 24–48 h is considered feasible using the same UPLC-QTOF method as STA and headspace gas chromatography.

The board-certified forensic pathologist evaluated whether EA-Triage could provide adequate information for determining the manner and/or COD and assess whether the case had nonsuspicious circumstances or required conventional autopsy. If possible, to determine the manner and/or COD, the board-certified forensic pathologist applied ICD-10 codes according to established guidelines. In cases where suspicion of a criminal act arose; the determination of the manner or COD remained unclear; the external examination or PMCT revealed injuries incongruent with the provided case history, e.g., unexpected trauma; the sQTA did not detect lethal intoxication by quantification; or if other factors of police interest, e.g., facial petechiae, were detected during the external examination, the board-certified forensic pathologist recommended complete medico-legal autopsy. For all cases, any unexplained case circumstances or suspicion of a criminal act led to a complete medico-legal autopsy regardless of the outcome of the sQTA or PMCT. The remaining cases in this study were referred to as cases with nonsuspicious circumstances.

### Statistics

Data were collected and managed using a database from REDCap electronic data capture tools hosted at the Department of Forensic Medicine, AU [20]. Statistical analysis was carried out using RStudio 2023.06.2 + 561 [21]. Categorical values are presented as percentages, and normally distributed continuous values are presented as means. Sensitivity and specificity were calculated using the ICD-10 code for COD from EA-Triage as the index and conventional autopsy as the gold standard for reference using epiR, RStudio. Only the ICD-10 codes related to the underlying COD were matched with the original codes from the medico-legal autopsy reports (Online Resource 3).

## Results

### Characteristics of identified drug-related deaths

**Fig. 1:**
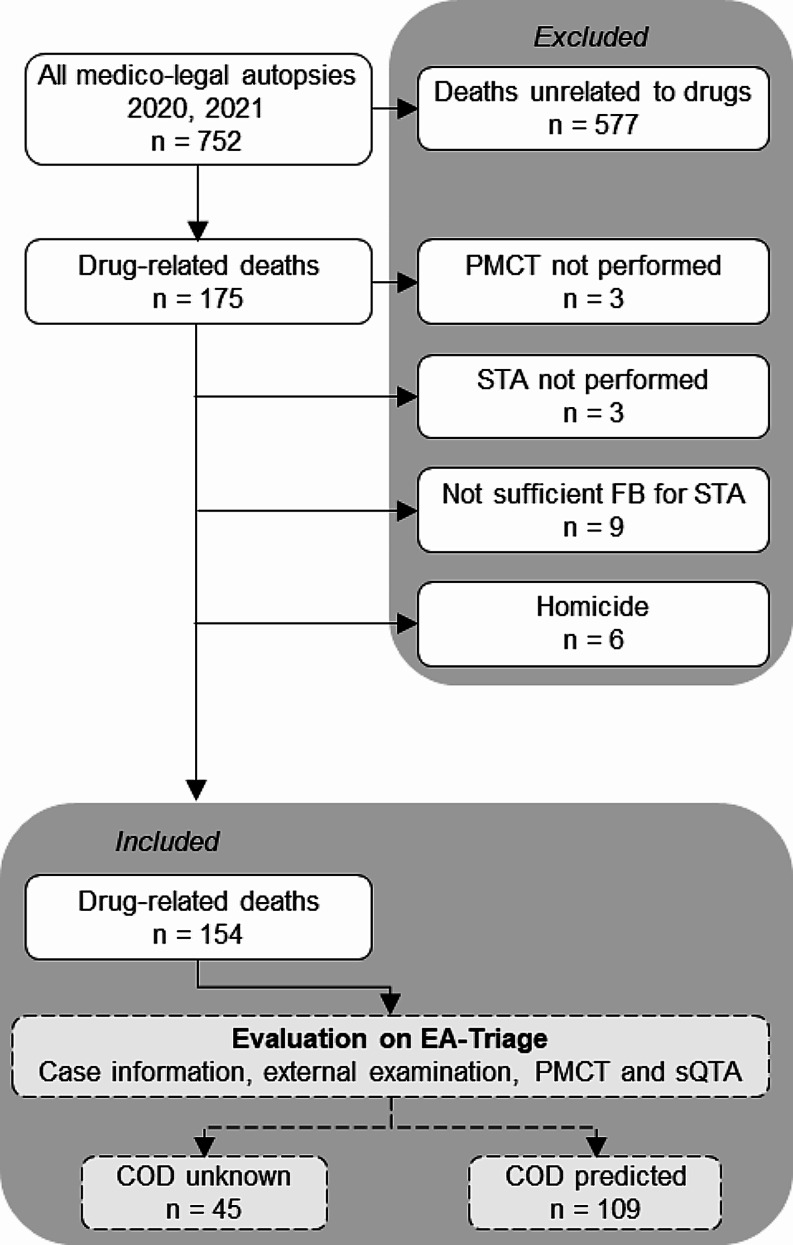
Flowchart of identified cases according to the inclusion criteria and evaluation by enhanced autopsy triage (EA-Triage) by a board-certified forensic pathologist in the determination of cause of death (COD). Postmortem computed tomography (PMCT) was not performed in three cases, one due to technical issues, one because the body exceeded the scanning capacity, and one because of the prioritization of resources during the COVID-19 pandemic. Systematic toxicological analysis (STA) was not required for three cases. In nine cases, femoral blood (FB) was not available due to decomposition. COD: Cause of death, PMCT: postmortem computed tomography, STA: Systematic toxicologcal analysis, sQTA: simulated quick toxicological analysis, FB: femoral blood

We identified 154 drug-related deaths that underwent medico-legal autopsies, including PMCT and STA, between 2020 and 2021 at the Department of Forensic Medicine, AU (Fig. 1). In 71% (*n* = 109) of the drug-related deaths, a COD could be predicted using EA-Triage. The average postmortem interval (time between death or discovery of death and the initiation of autopsy) was 3.5 days due to the selection process prior to autopsy, ranging from being performed on the same day to a maximum of 8 days.

The mean age at death was 40.6 years, and 82% (*n* = 126) were males (Table 1). The most common manner of death for drug-related deaths according to medico-legal autopsy was accidental (55%, *n* = 85). A diagnosis of at least one psychiatric disorder was present in 54% (*n* = 82), most commonly depression or schizophrenia. The majority of cases had a history of drug use of opioids, cocaine, or other psychoactive substances (95%, *n* = 146). The remaining cases had no past or current history of drug use, but three cases of isolated cannabis use and four cases of illegal drug use were detected on the STA. Severe decomposition occurred in only 5% of the samples; however, it was still possible to extract FB for the STA during the medico-legal autopsy. In 46% (*n* = 68) of cases, resuscitation involving manual chest compression, a chest compression system (LUCAS), medical treatment or defibrillation was performed. Facial petechiae were present in 20% of cases at the time of the external examination.

**Table 1:** Case characteristics of drug-related deaths

*n* (%)	154
Gender	
Male	126 (81.8)
Female	28 (18.2)
Age (mean(range))	40.6 (range 17–70 years)
Manner of death	
Accidental	85 (55.2)
Natural	29 (18.8)
Suicide	14 (9.1)
Undetermined	26 (16.9)
Psychiatric disorder^a^	83 (53.9)
Depression	22 (26.5)
Schizophrenia	19 (22.9)
Other psychiatric disorders^b^	52 (62.7)
Decomposition	
None or mild	132 (85.7)
Moderate	15 (9.7)
Severe	7 (4.5)
Resuscitation	68 (45.6)
Facial petechiae	31 (20.3)

^a^ Rely on ICD-10 coding, one case could have more than one psychiatric disorder simultaneously.

^b^ Covers bipolar disorder, posttraumatic stress disorder (PTSD) and attention deficit hyperactive disorder (ADHD) as the most common but also unspecified psychiatric diagnosis from the medical history.

### Cause of death agreement

The overall agreement between the COD determined by EA-Triage and the COD obtained from conventional autopsy was 73% (*n* = 113), including cases for which the COD was unknown (Table 2). Conventional autopsy reported a finding of unknown COD in 3% (*n* = 5) cases, but for EA-Triage, the COD was unknown in 29% (*n* = 45) of cases. The cases with an unknown COD according to EA-Triage were distributed according to manner of death at the medico-legal autopsy as follows: 51% (*n* = 23) were natural, 27% (*n* = 12) were accidental, 7% (*n* = 3) were suicides and 16% (*n* = 7) were unknown. In cases of fatal intoxication with drugs, the agreement between COD determined by EA-Triage and conventional autopsy was 92% (*n* = 97).

**Table 2:** Cross table on the cause of death determined by a board-certified forensic pathologist based on enhanced autopsy triage (EA-Triage) with integrated PMCT and sQTA compared to medico-legal autopsy as the gold standard

			EA-Triage																		
Cause of death			Intoxication					External conditions						Disease							
			Drugs	Volatiles	Alcoholic ketoacidosis	Diabetic ketoacidosis	Other	Firearm	Fire	Hanging	Traffic fatality	Exposure	Surgical malpractice	Cardiac	DVT	Pulmonary	Gastrointestinal	Infection	Cancer	**Unknown**	**Total**
**Autopsy**	**Intoxication**	Drugs	**97**	0	0	0	0	0	0	0	0	0	0	0	0	0	0	0	0	9	106
		Volatiles	0	**0**	0	0	0	0	0	0	0	0	0	0	0	0	0	0	0	2	2
		Alcoholic ketoacidosis	0	0	**2**	0	0	0	0	0	0	0	0	0	0	0	0	0	0	0	2
		Diabetic ketoacidosis	0	0	0	**3**	0	0	0	0	0	0	0	0	0	0	0	0	0	1	4
		Other	0	0	0	0	**0**	0	0	0	0	0	0	0	0	0	0	0	0	1	1
	**External conditions**	Firearm	0	0	0	0	0	**2**	0	0	0	0	0	0	0	0	0	0	0	0	2
		Fire	0	0	0	0	0	0	**2**	0	0	0	0	0	0	0	0	0	0	2	4
		Hanging	0	0	0	0	0	0	0	**1**	0	0	0	0	0	0	0	0	0	2	3
		Traffic fatality	0	0	0	0	0	0	0	0	**1**	0	0	0	0	0	0	0	0	2	3
		Exposure	0	0	0	0	0	0	0	0	0	**0**	0	0	0	0	0	0	0	1	1
		Surgical malpractice	0	0	0	0	0	0	0	0	0	0	**0**	0	0	0	0	0	0	1	1
	**Disease**	Cardiac	0	0	0	0	0	0	0	0	0	0	0	**0**	0	0	0	0	0	11	11
		DVT	0	0	0	0	0	0	0	0	0	0	0	0	**0**	0	0	0	0	1	1
		Pulmonary	0	0	0	0	0	0	0	0	0	0	0	0	0	**0**	0	0	0	3	3
		Gastrointestinal	0	0	0	0	0	0	0	0	0	0	0	0	0	0	**0**	0	0	2	2
		Infection	1	0	0	0	0	0	0	0	0	0	0	0	0	0	0	**0**	0	1	2
		Cancer	0	0	0	0	0	0	0	0	0	0	0	0	0	0	0	0	**0**	1	1
		**Unknown**	0	0	0	0	0	0	0	0	0	0	0	0	0	0	0	0	0	**5**	5
		**Total**	98	0	2	3	0	2	2	1	1	0	0	0	0	0	0	0	0	45	154

DVT: Deep vein thrombosis

The board-certified forensic pathologists were confident in their prediction of COD by EA-Triage in 109 cases, excluding cases of unknown COD (Fig. 1). The agreement between the COD obtained by EA-Triage and that determined by conventional autopsies was 99% (*n* = 108) without unknown CODs. The COD in the remaining case was a combination of sepsis and lethal intoxication.

### Recommendation for medico-legal autopsy

**Fig. 2:**
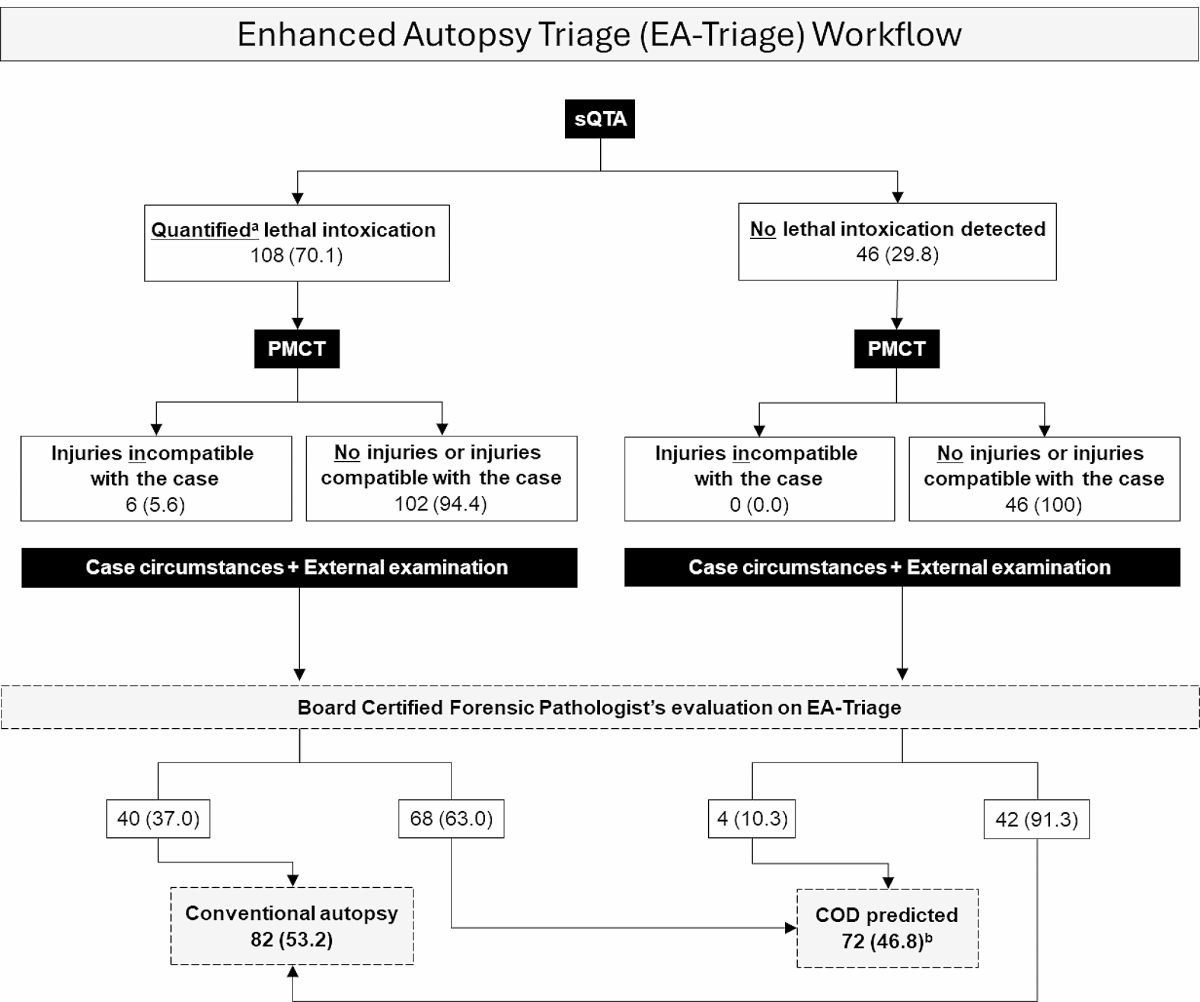
Workflow of the enhanced autopsy triage (EA-Triage) setup with integrated postmortem computed tomography (PMCT) and simulated quick toxicological analysis (sQTA) and the corresponding cases of drug-related deaths. Case circumstances could change the outcome independent of the ability of EA-Triage to provide a cause of death (COD). All cases were evaluated using EA-Triage by one of two board-certified forensic pathologists. PMCT findings were categorized as injuries compatible or incompatible with the case. Consequently, each individual injury was assessed in accordance with the case circumstances and medical history for compatibility, e.g., resuscitative efforts. Cases are displayed as n (%) ^a^ Quantification included toxicological evaluation of ethanol, carbon monoxide and 88 selected drugs and metabolites (Online Resource 2) ^b^ Cases with nonsuspicious circumstances where the COD could be determined through EA-Triage

Overall, 53% (*n* = 82) of the cases were recommended for complete medico-legal autopsy due to suspicious circumstances, the presence of facial petechiae, malpractice suspicion, police involvement at the time of death, or qualitative detection of drugs with no quantification results in sQTA. The remaining cases (*n* = 72, 47%) were considered nonsuspicious circumstances. In these cases, the board-certified forensic pathologists felt confident in answering all forensic aspects of the case based on EA-Triage. However, in one case, the conclusion reached by EA-Triage was intoxication, whereas sepsis and intoxication with prescription drugs was the COD according to the medico-legal autopsy. The workflow of the board-certified forensic pathologists and the corresponding cases according to the EA-Triage are depicted in the flowchart below (Fig. 2).

Among the 72 cases with nonsuspicious circumstances and a predicted COD, 85% (*n* = 61) were males, and the mean age was 40.4 years (range 18–67 years). The manner of death, as determined by both medico-legal autopsy and EA-Triage, was accidental in 58%/57%, natural in 8%/7%, suicidal in 11%/10% and undetermined in 22%/26%, respectively.

### Quick toxicological analysis

According to conventional autopsy, the most frequent COD in drug-related deaths was intoxication (75%, *n* = 115). The quantitative analysis of the sQTA correctly revealed lethal concentrations in 94% (*n* = 108) of these deaths (Fig. 2). The qualitative analysis of the sQTA showed the presence of lethal compounds in 6% (*n* = 7) of the intoxication deaths, necessitating further analysis to determine the COD. The substances detected in the qualitative analysis were one case of insulin, two cases of volatiles and one case with isotonitazene. In the last three cases, sQTA partly detected lethal intoxication, because these cases had combinations of 3–5 different substances. The missing substances were flubromazolam, hydroxyzine and isopropanol.

### Cause of death agreement in cases with nonsuspicious circumstances

In cases with nonsuspicious circumstances, EA-Triage and conventional autopsy agreed on COD in 99% (*n* = 71). The most common COD was intoxication with drugs (*n* = 62). Sensitivity and specificity were calculated for the COD determined by EA-Triage compared to that determined by medico-legal autopsy. The results are displayed in Table 3.

**Table 3:** Sensitivity and specificity for cause of death (COD) determined by enhanced autopsy triage (EA-Triage) regarding the underlying COD in drug-related deaths with nonsuspicious circumstances (*n* = 72). Medico-legal autopsies was used as a reference standard

Cause of death	EA-Triage			
	Sensitivity		Specificity	
	TP/P	% (95% CI)	TN/N	% (95% CI)
Intoxication^a^ (*n* = 62)	62/62	100% (0.94-1.00)	9/10	90% (0.55-1.00)
Diabetic ketoacidosis (*n* = 3)	3/3	100% (0.29-1.00)	69/69	100% (0.95-1.00)
Alcoholic ketoacidosis (*n* = 2)	2/2	100% (0.16-1.00)	70/70	100% (0.95-1.00)
Traffic fatality (*n* = 1)	1/1	100% (0.02-1.00)	71/71	100% (0.95-1.00)
Fire exposure (*n* = 1)	1/1	100% (0.02-1.00)	71/71	100% (0.95-1.00)
Firearm (*n* = 1)	1/1	100% (0.02-1.00)	71/71	100% (0.95-1.00)
Hanging (*n* = 1)	1/1	100% (0.02-1.00)	71/71	100% (0.95-1.00)

TP: True positive, P: Total number of positives from reference standard, TN: True negative, N: Total number of negatives from reference standard, CI: Confidence interval

^a^ Intoxication with illegal and prescription drugs

## Discussion

Drug-related deaths constitute a large proportion of forensic cases in Denmark because current legislation requires routine autopsies in these deaths. This study showed that this subgroup of deaths could be eligible for COD prediction based on EA-Triage including PMCT and QTA. Notably, EA-Triage and conventional autopsy agreed on COD in 99% of drug-related deaths with nonsuspicious circumstances.

Many countries, including Denmark, which has the lowest autopsy rate among the Nordic countries, are experiencing declining autopsy rates [22]. The reasons for this decline are most likely a combination of prioritizing resources; religious, cultural or emotional reluctance against invasive autopsy; variable legislation; and biosafety awareness [23]. To accommodate this trend, an increasing need for alternatives to autopsies has emerged. Therefore, the incorporation of minimally invasive screening techniques in the forensic field has been widely researched for different subgroups. In other investigations on preautopsy COD determination, toxicological analysis has been shown to improve the diagnostic agreement. Hueck et al. increased the agreement on COD from 85.9 to 95.3% when considering histological and toxicological findings in unnatural deaths [11]. Leth et al. also had toxicological analysis available on large amounts of material from the Danish forensic population [12]. Consistent with our results, they found that in 50% of cases with intoxication, a COD could have been predicted if a day-to-day toxicological service combined with PMCT had been available. In a study from New Mexico, cases of drug-related deaths were examined to evaluate the ability to determine COD prior to autopsy when considering PMCT and toxicology analysis. The COD agreement was 78%, but it was higher for individuals younger than 50 years (84%) [3]. Hence, our study results are consistent with prior research. Overall, we demonstrated a substantial agreement of 73% (99% when excluding cases with unknown COD) for cases without suspicious circumstances between EA-Triage and conventional autopsy in determining COD in drug-related deaths when toxicological analysis is considered.

In other subgroups, the use of EA-Triage might be more complex. Previous studies on the use of PMCT for nontraumatic deaths have shown poor diagnostic accuracy. Femia et al. reported only 35% agreement on COD in unexplained deaths between PMCT and conventional autopsy [24]. PMCT was considered beneficial only for neurological, traumatic, and gastrointestinal deaths. We also found that EA-Triage had a greater proportion of patients with an unknown COD (29%) than did medico-legal autopsy (3%). Cases with an unknown COD were mostly natural deaths due to disease, suggesting that in this subgroup, it could be difficult to predict a COD based only on EA-Triage. Therefore, other screening techniques could be considered relevant to preautopsy investigations for natural deaths. Femia et al. also showed that postmortem computed magnetic resonance (PMMR) identified COD in 50% of suspected cardiac and neurological deaths compared to 35% identified by PMCT [24]. In another study of cardiovascular deaths, compared with unenhanced PMCT, contrast-enhanced PMCT increased the agreement on COD from 65 to 95% [25].

Drug-related deaths are frequently nontraumatic on PMCT, and lethal concentrations of drugs is detected in postmortem blood. PMCT can be used to rule out injuries and serve as a screening tool to exclude any traumatic involvement incompatible with the case circumstances. Additionally, resuscitative efforts, e.g., rib fractures compatible with the case circumstances, can be identified. Combined with QTA, which can detect and quantify the most common lethal intoxications within 24–48 h, the ability to determine COD was comparable to that of conventional autopsies in this subgroup. Thus, EA-Triage has the possibility to accurately predict COD prior to autopsy in selected subgroups. This approach could guide police investigations at an early stage, minimize the burden of family members and offer an alternative in cases where autopsy is refused, e.g., those where it is prohibited by religious beliefs.

sQTA identified most lethal intoxications in the present study (94%). However, it was not suitable for cases with suspicion of the use of volatiles, designer drugs or uncommon drugs. Volatiles require further analysis upon request based on circumstances, and the remaining drugs would be detected in the qualitative screening. In one case of suspected intoxication with insulin, neither sQTA nor STA would have been able to detect exogenous insulin, and the COD was determined from case circumstances with strong indications of excess insulin intake. QTA was originally designed for clinical purposes in the emergency department, where it is acceptable that reliability can be compromised if treatment is necessary for preventing death. QTA in a forensic setting requires high reliability because the results must be valid in a legal setting. Standard toxicological analysis always includes verification of the screening results with a different technique, meaning that behind a reported result, there are two different analyses carried out by different technicians, usually on different subsamples. This procedure lowers the risk of sample mix-up and the reporting of false-positive results, which should not be neglected as a limitation of QTA. Simultaneous testing of postmortem urine, cardiac blood or vitreous humor as well as femoral blood could verify the analytical results and toxicological assessment, increasing the reliability of QTA. The sampling of postmortem FB can be challenging due to decomposition and low blood volume. The technique is well known from studies on PMCT angiography but will not be further addressed here [26]. However, to ensure that all forensic concerns are met, there should always be recommendations for medico-legal autopsy in cases without sufficient material or quantitative results explaining the COD.

EA-Triage may contribute substantial value to preautopsy COD determination in the subgroup of drug-related deaths. The agreement between EA-Triage and conventional autopsy for cases with nonsuspicious circumstances was 99%. The discrepancy in COD arose in one case of competing CODs. This case was determined to be intoxication with prescription drugs by EA-Triage; however, the medico-legal autopsy microbiology analysis revealed sepsis. EA-Triage missed this competing COD and potentially other natural CODs, such as certain cardiovascular events, uncommon intoxications, and other bacterial infections, if there was no history to suggest this possibility. In cases with no indication for further examination, there is also a risk of incorrect determination of COD during conventional autopsy. For drug-related deaths, competing CODs rarely occur. However, the expansion of EA-Triage to other groups at the medicolegal inquest could improve the selection for medico-legal autopsy. Some cases may not need an autopsy according to national legislation and guidelines. In others, the possibility arises that EA-Triage results would determine an unforeseen need for autopsy, prompting selection of the “proper” forensic population for conventional autopsy. Retrospective studies, such as this one, are the first step toward implementing triage of the complete forensic population. Implementation of EA-Triage will be possible once QTA has been validated in a forensic setting. The anticipated challenges of implementation include logistics and geographic considerations, as well as the likelihood of additional costs and workload. These factors should be taken into account during the implementation phase.

When comparing the experience of implementing screening tools prior to autopsy, generalization should be performed with caution. The use of different populations and selection methods for autopsies limits the ability to compare countries. At the Department of Forensic Medicine, AU, one-third of medico-legal autopsies are intoxication cases, but in other countries, firearm fatalities and homicides may be more prevalent. Although our findings support previous research on the high agreement in COD when comparing PMCT and QTA with conventional autopsy for drug-related deaths, this agreement might not be as high for other subgroups of the Danish population or other forensic populations. Further research and experience in this area are needed to determine the accuracy of COD determination obtained from conventional autopsies.

Limitations of our study included the selection of drug-related cases. It would have been optimal to have prospectively selected cases in cooperation with the deciding police officer at the current medico-legal inquest. Instead, the study group was based on an in-house coding system of drug users, and since the coding was registered after the results of the STA, some cases may have been irrelevant in a prospective scenario (e.g., traumatic deaths). Another limitation of the retrospective study design was the variation in case material available from ongoing cases during a medico-legal autopsy compared to the summarized material presented at the EA-Triage setup. This resulted in fewer details in ICD-10 coding compared to the original COD coding from the medico-legal autopsy. A conservative diagnostic approach was applied because any suspicious case circumstances, regardless of the results from EA-Triage, led to recommendations for medico-legal autopsies. This likely led to an overestimation of suspicious cases recommended for medico-legal autopsy. However, a conservative approach must be advised in this stage of research. Testing the ability of EA-Triage to assess manner of death was not the focus of this study; however, determination of manner of death is important to close a forensic case without autopsy.

## Conclusion

In conclusion, board-certified forensic pathologists effectively used the EA-Triage setup to predict the COD in drug-related cases, with 73% overall agreement with conventional autopsies. Cases with nonsuspicious circumstances, accounting for 47% of the drug-related deaths, showed 99% agreement with autopsy results when a COD was predicted. The QTA detected the most common lethal intoxications, but limitations in the QTA setup must be addressed and validated. EA-Triage relies on a conservative diagnostic approach and the case information available from the police investigation, medical history, and pre-hospital services to minimize that competing CODs are not overlooked. Generalizing our findings to other countries must be done with caution, considering the existing legislation and selection criteria. While EA-Triage showed promise in guiding early police investigations, further research, particularly prospective studies, is needed to validate its accuracy and feasibility in a real-life forensic setting before deployment.

## Key points

1. EA-Triage has a high diagnostic accuracy regarding COD comparable to that of conventional autopsy.

2 Individuals who have died from past or current use of illegal drugs are suitable for EA-Triage, given that this subgroup is often nontraumatic on PMCT and involves lethal intoxication with common drugs.

3 Implementing EA-Triage can aid in guiding police investigations at an early stage and minimize the burden of family members.

4 EA-Triage is ready for implementation when the QTA has been validated in a forensic setting

5 Further prospective research is needed to validate and generalize the accuracy of EA-Triage regarding COD.

## Electronic supplementary material

Below is the link to the electronic supplementary material.

Supplementary Material 1

Supplementary Material 2

Supplementary Material 3
