# Supplementary material for: Enhanced autopsy triage (EA-Triage) in drug-related deaths: integrating quick toxicological analysis and postmortem computed tomography
Source: Forensic Sci Med Pathol. 2024 Apr 29;21(1):229–38. doi: 10.1007/s12024-024-00819-2 (PMC11953209; doi:10.1007/s12024-024-00819-2)
Supplement: Supplementary file 1 — Supplementary Material 1 [file 12024_2024_819_MOESM1_ESM.pdf]

Enhanced Autopsy Triage (EA-Triage) in Drug-Related Deaths: Integrating Quick Toxicological Analysis and Postmortem Computed Tomography, Forensic Science, Medicine, and Pathology

**Online Resource 1:** Standardized CT description template; Detailed review of all organs divided into sections, as well as an assessment of both skeleton and soft tissues		
Case number	Name	Social Security number

CT description	Forensic radiographer / Board certified forensic pathologist	Radiologist
**Head**<br>Skeletal: ☐ NAD<br>Soft tissue: ☐ NAD		
**Neck**<br>Skeletal: ☐ NAD<br>Soft tissue: ☐ NAD		
**Thorax**<br>Soft tissue: ☐ NAD		
**Abdomen**<br>Soft tissue: ☐ NAD		
**Pelvis**<br>Soft tissue: ☐ NAD		
**Axial skeleton**<br>Thorax: ☐ NAD<br>Columna: ☐ NAD<br>Pelvis: ☐ NAD		
**Upper extremities**<br>Skeletal: ☐ NAD<br>Soft tissue: ☐ NAD		
**Lower extremities**<br>Skeletal: ☐ NAD<br>Soft tissue: ☐ NAD		

NAD: No abnormalities detected

**Costa fractures**						
**DXT**				**SIN**		
Front	Lateral	Back	Costa	Back	Lateral	Front
			**1**			
			**2**			
			**3**			
			**4**			
			**5**			
			**6**			
			**7**			
			**8**			
			**9**			
			**10**			
			**11**			
			**12**			

DXT: dexter, Sin: sinister, X: Fracture (new), Seq: Fracture sequelae, Callus: Fracture with callus formation

Date:	Signature:
