# Supplementary material for: Enhanced autopsy triage (EA-Triage) in drug-related deaths: integrating quick toxicological analysis and postmortem computed tomography
Source: Forensic Sci Med Pathol. 2024 Apr 29;21(1):229–38. doi: 10.1007/s12024-024-00819-2 (PMC11953209; doi:10.1007/s12024-024-00819-2)
Supplement: Supplementary file 2 — Supplementary Material 2 [file 12024_2024_819_MOESM2_ESM.pdf]

**Enhanced Autopsy Triage (EA-Triage) in Drug-Related Deaths: Integrating Quick Toxicological Analysis and Postmortem Computed Tomography,** Forensic Science, Medicine, and Pathology

**Online Resource 2:** List of the 88 drugs and metabolites included in the quantification of the QTA together with ethanol and carbon monoxide. All quantified results gave a postmortem concentration.	
Agomelatin	Lysergic acid diethylamide (LSD)
Alprazolam	3,4-Methylenodioxymethamphetamine (MDMA)
Amfetamin	Melatonin
Amitriptylin	Metamfetamin
Amlodipin	Metformin
Aripiprazol	Methadon
Atenolol	Methylphenidat
Baclofen	Metoclopramide
Benzoylecgonin	Metoprolol
Beta-hydroxybutyrate	Metotrexat
Bisoprolol	Mianserin
Bromazepam	Midazolam
Buprenorphin	Mirtazapine
Carbamazepine 10,11-epoxide	Morphine
Carvediol	Morphin-6-beta-D-glucuronid
Chlordiazepoxid	Nitrazepam
Chlorprothixen	Norclomipramin
Citalopram (+ escitalopram)	Nordazepam
Clomipramin	Nortriptyline
Clonazepam	Olanzapine
7-amino-clonazepam	Orphenadrine
Clozapin	Oxazepam
Cocain	O-Demethyltramadol
Codein	O-Demethylvenlafaxin
Cyclizin	Oxycodone
Dehydroaripiprazol	Paliperidon
Demoxepam	Paracetamol
Desimipramin	Paroxetine
Diazepam	Phenobarbital
Duloxetine	Pregabalin
Felodipine	Promethazine
Fentanyl	Propranolol
Fluoxetin	Quetiapine
Gabapentin	Risperidone
GHB	Salicylsyre
Haloperidol	Sertraline
Ibuprofen	THC-COOH-glucuronid
Imipramine	Tramadol
Ketamin	Venlafaxine
Ketobemidone	Verapamil
Lamotrigine	Vortioxetine
Lercanidipine	Zolpidem
Levetiracetam	Zopiclon
Levomepromazine	Zuclopenthixol
