# Supplementary material for: Enhanced autopsy triage (EA-Triage) in drug-related deaths: integrating quick toxicological analysis and postmortem computed tomography
Source: Forensic Sci Med Pathol. 2024 Apr 29;21(1):229–38. doi: 10.1007/s12024-024-00819-2 (PMC11953209; doi:10.1007/s12024-024-00819-2)
Supplement: Supplementary file 3 — Supplementary Material 3 [file 12024_2024_819_MOESM3_ESM.pdf]

Enhanced Autopsy Triage (EA-Triage) in Drug-Related Deaths: Integrating Quick Toxicological Analysis and Postmortem Computed Tomography, Forensic Science, Medicine, and Pathology

Online Resource 3: ICD-10 code for underlying COD categorization adjusted for manner of death

**COD category**		**ICD-10 code**	**ICD-10 code description**
Intoxication with illegal and prescription drugs	Intoxication by and exposure to narcotics and psychodysleptics [hallucinogens]	**X42**	Accidental poisoning by and exposure to narcotics and psychodysleptics [hallucinogens], not elsewhere classified
	Intoxication by and exposure to narcotics and psychodysleptics [hallucinogens]	**X62**	Intentional self-poisoning by and exposure to narcotics and psychodysleptics [hallucinogens], not elsewhere classified
	Intoxication by and exposure to narcotics and psychodysleptics [hallucinogens]	**Y12**	Poisoning by and exposure to narcotics and psychodysleptics [hallucinogens], not elsewhere classified, undetermined intent
	Intoxication by and exposure to other and unspecified drugs, medicaments, and biological substances	**X44**	Accidental poisoning by and exposure to other and unspecified drugs, medicaments, and biological substances
	Intoxication by and exposure to other and unspecified drugs, medicaments, and biological substances	**X64**	Intentional self-poisoning by and exposure to other and unspecified drugs, medicaments, and biological substances
	Intoxication by and exposure to other and unspecified drugs, medicaments, and biological substances	**Y14**	Poisoning by and exposure to other and unspecified drugs, medicaments and biological substances, undetermined intent
Diabetic ketoacidosis		**E141**	Unspecified diabetes mellitus with ketoacidosis
Alcoholic ketoacidosis		**F101 + E872**	F101: Mental and behavioral disorders due to use of alcohol: harmful use
			E872: Unspecified acidosis
Firearms		**X72**	Intentional self-harm by handgun discharge
Hanging		**X70**	Intentional self-harm by hanging, strangulation and suffocation
Fire exposure		**X00**	Exposure to uncontrolled fire in building or structure
		**X09**	Exposure to unspecified smoke, fire and flames
Traffic fatality		**X81**	Intentional self-harm by jumping or lying before moving object
